# Re-mating across years and intralineage polygyny are associated with greater than expected levels of inbreeding in wild red deer

**DOI:** 10.1111/j.1420-9101.2012.02626.x

**Published:** 2012-10-05

**Authors:** K V Stopher, D H Nussey, T H Clutton-Brock, F Guinness, A Morris, J M Pemberton

**Affiliations:** * Institute of Evolutionary Biology, University of EdinburghEdinburgh, UK; † James Hutton (formerly Macaulay) InstituteCraigiebuckler, Aberdeen, UK; ‡ Centre for Immunity, Infection and Evolution, University of EdinburghEdinburgh, UK; § Department of Zoology, University of CambridgeCambridge, UK

**Keywords:** *Cervus elaphus*, dispersal, genetic structure, inbreeding avoidance, philopatry, relatedness

## Abstract

The interaction between philopatry and nonrandom mating has important consequences for the genetic structure of populations, influencing co-ancestry within social groups but also inbreeding. Here, using genetic paternity data, we describe mating patterns in a wild population of red deer (*Cervus elaphus*) which are associated with marked consequences for co-ancestry and inbreeding in the population. Around a fifth of females mate with a male with whom they have mated previously, and further, females frequently mate with a male with whom a female relative has also mated (intralineage polygyny). Both of these phenomena occur more than expected under random mating. Using simulations, we demonstrate that temporal and spatial factors, as well as skew in male breeding success, are important in promoting both re-mating behaviours and intralineage polygyny. However, the information modelled was not sufficient to explain the extent to which these behaviours occurred. We show that re-mating and intralineage polygyny are associated with increased pairwise relatedness in the population and a rise in average inbreeding coefficients. In particular, the latter resulted from a correlation between male relatedness and rutting location, with related males being more likely to rut in proximity to one another. These patterns, alongside their consequences for the genetic structure of the population, have rarely been documented in wild polygynous mammals, yet they have important implications for our understanding of genetic structure, inbreeding avoidance and dispersal in such systems.

## Introduction

The use of molecular techniques to assign parentage in wild populations has been revolutionary in a variety of wild vertebrate taxa in revealing fine-scale spatial genetic structure arising from limited dispersal (Hughes, 1998; Piertney *et al*., 1999; Shorey *et al*., 2000; Nussey *et al*., 2005). In polygynous mammals, it is most common for females to be philopatric and males to disperse, leading to aggregations of females in matrilineal groups (Clutton-Brock, 1989). Where females are philopatric, and mating is nonrandom, it can have substantial effects on kinship and inbreeding within groups, particularly if males are also philopatric during breeding (Chesser, 1991). Recently, there has been a rise in the number of studies reporting a lack of inbreeding avoidance in vertebrate taxa and even inbreeding preference in some populations (for example: Kleven *et al*., 2005; Rioux-Paquette *et al*., 2010; Wang & Lu, 2011). However, undertaking robust tests of inbreeding avoidance in wild populations is extremely challenging (Part, 1996; Keller & Arcese, 1998) and requires careful consideration of the set of potential mates available. Further, it is constructive to understand what aspects of the mating system – such as spatial genetic structuring – are associated with such outcomes.

Two aspects of mating systems have been revealed in polygynous mammals which, although extremely rarely reported, are potentially highly significant to the link between philopatry and co-ancestry/inbreeding. These are ‘mate fidelity’ (or ‘re-mating frequency’), females mating with the same male in one or more distinct breeding attempts (but without the establishment of a pair bond, that is, in contrast to the permanent pair bonds exhibited in monogamous species), and ‘intralineage polygyny’, whereby female relatives show a propensity to mate with the same male (Rossiter *et al*., 2005). Despite the clear potential for such behaviour in species with female philopatry, few studies report having capitalized upon new molecular techniques to test for such behaviour in polygynous systems, and so the extent to which it occurs is difficult to assess. However, there is evidence that it occurs in three polygynous mammals: red deer, grey seals and greater horseshoe bats. In red deer, *Cervus elaphus*, estimates of re-mating rates from behavioural observations of a small number of breeding hinds (29–32) over two consecutive 2-year periods ranged from 15.6% to 24.1% (Clutton-Brock *et al*., 1982b). In a study of paternity in grey seals, *Halichoerus grypus*, 30% of pups born to the same mother were found to be in full-sibs. In a species where litter size is usually one, and in which females mate in multiple years, this suggests many females were re-mating with the same male across years (Amos *et al*., 1995). However, using a longer time series, the proportion of full-sibs was later estimated to be substantially lower in the same population (Worthington Wilmer *et al*., 2000), and no evidence of re-mating was found in a population of harbour seals (Coltman *et al*., 1999). Other evidence for re-mating has been found in the greater horseshoe bat, *Rhinolophus ferrumequinum* (Rossiter *et al*., 2005), in which the authors showed 56.8% of females mating in more than 1 year paired with the same male in multiple years, and that such repeated pairings between individuals occurred more than expected by chance.

Further to evidence for re-mating, molecular studies of the greater horseshoe bat have revealed that matrilineal relatives also mated with the same males more frequently than expected by chance (termed ‘intralineage polygyny’). Intralineage polygyny is expected to arise when there are both strong philopatry amongst females – so that females are likely to associate in kin groups – and also strong polygyny, so that those groups of females are likely to be monopolized by single males; however in the greater horseshoe bat, females also mate with males in satellite caves away from their natal site (Rossiter *et al*., 2005), and so this behaviour cannot entirely be explained by female philopatry. The interplay between philopatry and polygyny, resulting in intralineage polygyny, is likely to have important consequences for population genetic structure, increasing co-ancestry amongst females within social groups (Chesser, 1991). In the greater horseshoe bat, intralineage polygyny combined with females repeatedly pairing with particular males is associated with an increase in pairwise relatedness coefficients, and significant genetic differentiation between groups of matrilineal relatives (Rossiter *et al*., 2005).

Rossiter *et al*. (2005) argue that increased pairwise relatedness is likely to strengthen ties between roosting females and therefore cooperation within social groups. In general, where generations of females are overlapping, intralineage polygyny and females re-mating with previous partners may raise co-ancestry but also increase the potential for inbreeding to occur (Chesser, 1991; Storz, 1999). The extent to which these processes result in increased inbreeding coefficients will be dependent upon whether males show fidelity to mating sites between years, whether male tenure overlaps with the onset of sexual maturity of female offspring and whether there is random dispersal of male offspring, particularly whether male offspring ever obtain mating success within their natal group (Storz, 1999). In general, the risk of inbreeding is not increased by female philopatry unless there is also a nonrandom spatial distribution of males with respect to relatedness (Foerster *et al*., 2006). Further, even where demographic circumstances increase the potential for inbreeding, if individuals are able to recognize kin, they may avoid mating with them (Pusey & Wolf, 1996; Foerster *et al*., 2006), although there is little evidence for this in species with dispersal (Clutton-Brock & McAuliffe, 2009). In greater horseshoe bats, no increase in inbreeding was found from that expected under random mating (Rossiter *et al*., 2005).

### This study: the potential for re-mating and intralineage polygyny

In this study, we use molecular paternity data to examine the patterns of mating in a wild population of red deer living on the North Block of the Isle of Rum, Scotland. We quantify the extent to which females mate with the same male in multiple years and to which females from the same matriline tend to mate with the same male. Further, we examine associated changes in pairwise relatedness and inbreeding within the population. Testing whether females re-mating with previous partners and intralineage polygyny are occurring more than would be expected by chance, and for the effects of such parameters on relatedness and inbreeding coefficients, necessarily requires comparing the observed mating outcomes with those expected under random mating, which can be modelled using simulated data. Such techniques can also be used to determine whether the observed outcomes are an artefact of known aspects of the breeding system, such as a preference for particular mating sites, by modelling such information within the simulations. This method of pedigree simulation, incorporating assumptions about mate availability and spatial parameters, has been successfully used to assess whether inbreeding avoidance occurs more often than expected under random mating (Keller & Arcese, 1998; Hansson *et al*., 2007; Szulkin *et al*., 2009); yet to date, studies examining pairs re-mating have relied on somewhat anecdotal evidence to suggest that the findings are not an outcome of site fidelity (Amos *et al*., 1995; Rossiter *et al*., 2005).

Red deer have a polygynous, harem defence mating system, in which males compete to herd and defend groups of females, and to mate with females within those groups which are in oestrus. Previous studies using behavioural data have found some evidence for both females re-mating with the same male (see above) and for intralineage polygyny in this system, with around 15% of daughters mating with the same male as their mother and 10% of mature sisters mating with the same male (Clutton-Brock *et al*., 1982a,b). Various spatial and temporal aspects of the red deer mating system suggest the potential for both re-mating and intralineage polygyny. Males live outside the study area for the majority of the year, returning prior to the breeding season (rut) to the main hind feeding grounds to mate. Young males disperse from their natal groups after the age of 2, and outside the rut, adult males do not show spatial genetic structure (Clutton-Brock *et al*., 1982a,b; Nussey *et al*., 2005). However, whether there is spatial genetic structuring of males during the rut, when they return to defend harems in the study area, is unknown. Preliminary analyses have suggested that a male's location during the rut is highly repeatable, with 50–70% of variance in male location explained by male identity (K.V. Stopher, T.H. Clutton-Brock & J. Pemberton, unpublished data), suggesting that males return to rut in the same area in multiple years. Females in this population are philopatric, usually remaining within the natal group to which they were born, so that the female population consists of mostly matrilineal groups which demonstrate strong location fidelity (Albon *et al*., 1992). Very fine-scale genetic structuring (< 100 m) has been shown amongst females (although this has declined over time, Nussey *et al*., 2005). During the rut, females occupy a constricted version of their normal home range (Clutton-Brock *et al*., 1982a,b). Overall, therefore, the potential for males and females to mate in the same location each year is high, as is the potential for female relatives to be mating in the same place.

There is also substantial individual consistency of rut timing that potentially promotes these behaviours in the population. Males generally do not rut for the entire breeding season, but at some point become exhausted and leave the rutting area; male rut start, and median and end dates have been shown to be highly repeatable within individuals (Clements *et al*., 2011). Females are in oestrus only briefly and usually mate only once (Clutton-Brock *et al*., 1982a,b). Although the majority of oestruses occur during a 2-week peak of the breeding season, they can be distributed over as much as 4 months. Female oestrus date has not been found to be particularly repeatable within individuals; however, given that parturition date is highly repeatable and the two are significantly correlated at both the phenotypic and genetic level (Clements *et al*., 2011), this finding is potentially confounded by the power available to detect repeatability. Further, nonlactating females that are closely associated within the same social group have been found to have synchronized oestruses (Iason & Guinness, 1985), and Clements *et al*. (2011) noted a significant sire effect on female oestrus date, suggesting that there may be consistent spatial differences in female oestrus date combined with fidelity of rutting sites by males across years. This therefore suggests female relatives associating within the same area or matrilineal group may be prone to mating at the same time, and are therefore more likely to mate with the same male.

In this study, we compare the observed mating outcomes, derived from a genetic pedigree, to those produced under a number of random mating scenarios, each with sequentially greater constraints: fully random mating (‘Random’), random mating temporally constrained by the timing of female oestrus (‘Temporal’), random mating temporally constrained and also spatially constrained to potential mates within 500 or 100 m (‘Spatial 500 m’ and ‘Spatial 100 m’), and finally temporally and spatially constrained random mating in which the probability of a male mating is dependent either upon his age (‘Age-corrected’) or lifetime breeding success [‘Breeding Success–corrected’ (‘BS-corrected’)]. We compare the frequency at which repeated pairings occur and the levels of intralineage polygyny, in the observed and simulated pedigrees, as well as relatedness and inbreeding coefficients, to determine the extent to which such nonrandom mating occurs and the effect it has on the relatedness structure of the population.

## Materials and methods

### Study system

Data were collected from a wild population of red deer, *C. elaphus*, resident in the North Block of the Isle of Rum, Scotland, which has been intensively studied since 1971. The study area comprises approximately 14% of the island area as a whole and between 15–25% of the deer on the island. In this study, we studied mating success during the ruts of 1971–2006. In this population, all individuals can be recognized, through either natural markings or artificial markings applied when individuals are captured at birth. Individuals are assigned to matrilines by tracing an individual's maternal line back to one female alive when the study began. Eighty-five matrilines exist, with a maximum of nine generations over the years used in this analysis. Necessarily, this assumes all females at the start of the study are unrelated; although this assumption is unlikely to be realistic, it is conservative with respect to intralineage polygyny, given that some apparently unrelated females mating with the same male will in fact share a maternal ancestor. During the rut, daily censuses are conducted which record the location (to the nearest 100 m) and identity of all females and all males which are defending harems of females. Female oestrus date can be calculated by backdating from the date of birth of subsequent offspring by 235 days (standard deviation = 5); we then assume that the female has conceived within this 11-day ‘oestrus window’ (Clutton-Brock *et al*., 1982a,b). Females produce one offspring per year, although not all females breed in each year. Females can conceive at the age of two; after the age of five, female fecundity is generally constant until it begins to decline at around 13 years (Nussey *et al*., 2009). Male annual breeding success (ABS) is highly skewed (Clutton-Brock *et al*., 1982a,b) and is strongly correlated with age (Nussey *et al*., 2009). Males rarely breed before 5, with ABS peaking at 8–10 years and then declining in later life (Nussey *et al*., 2009). Males therefore begin breeding much later in life and have a much shorter breeding tenure than females.

### Paternity assignment

Daily observations are made during the calving season (approximately 20th May to 30th June) to identify calving date for each female and monitor neonatal survival (Clutton-Brock *et al*., 1982a,b), and to catch calves and take tissue samples for genotyping. Other individuals not caught at birth are sampled from cast antlers, by chemical immobilization or post-mortem. Individuals born since 1991 were genotyped at up to 15 highly variable microsatellites; prior to this, individuals were genotyped at up to eight microsatellites. Paternities were assigned using the programs MasterBayes (Hadfield *et al*., 2006) and colony2 (Wang & Santure, 2009) with > 80% individual confidence, with preference given to assignments made by the MasterBayes program, and colony2 used to assign paternities where MasterBayes could not assign a father at 80% individual confidence (see Walling *et al*., 2010 for full details). The use of categorical pedigrees such as this is potentially misleading, as they do not explicitly incorporate the error around paternity assignments. Analysis was undertaken to address this potential problem (presented in Data S1), and we were able to demonstrate that it has no effect on our findings.

### Analysis

All analyses were carried out in R 2.8.1 (R Development Core Team 2008).

For each year, lists of candidate females (those which calved the following spring) and candidate males (those seen to hold a harem in that year) were drawn up, and six types of simulated pedigrees were generated:

1. ‘Random’: each female was randomly assigned a male from the candidate male list.
2. ‘Temporal Random’: each female was randomly assigned a male from the candidate male list that was known to have held a harem during her calculated ‘oestrus window’.
3. ‘Spatial Random’: as for temporal random, but the list of potential males was further restricted to those holding a harem within (i) 500 m (‘Spatial 500 m’) or (ii) 100 m (‘Spatial 100 m’) of the female's location on the potential day of conception. These values were chosen after preliminary analysis revealed that 75% of females mate with males rutting within 500 m of their location on the day of conception and 50% of females mate with a male within 100 m of their location.
4. ‘Age-corrected’: as for ‘Spatial 100 m’, but with the sampling of temporally and spatially available males weighted by the probability of gaining reproductive success given their age. Male ABS is highly correlated with age (Nussey *et al*., 2009). We constructed a linear model of age and its quadratic term against male ABS for the pedigree data used in this study (2083 observations across 603 males), and from this extracted the probability of males of different ages gaining a paternity. The sampling of candidate males was then weighted by this probability.
5. ‘BS-corrected’: as for ‘Spatial 100 m’, but with the sampling of temporally and spatially available males weighted by the probability of gaining reproductive success given the male's lifetime breeding success. To calculate this, we constructed a linear model of male identity against male ABS, and from this extracted the probability of each male gaining a paternity.

Candidate males within each year were sampled with replacement, and all females that calved in each year were assigned a new mate. Each randomization was constructed on an annual basis, but then for each randomization type, all years were combined to produce a randomized pedigree covering the whole study period. This was repeated 1000 times for each randomization type. Offspring retained their true mothers throughout the simulations.

### Pedigree statistics

Pedigree statistics (e.g. re-mating frequency, intralineage polygyny, pairwise relatedness and inbreeding coefficients) were calculated for each of the 1000 simulations of each randomization type. For each pedigree statistic, an average and standard deviation were calculated across the 1000 simulations.

All measures described were compared between the observed pedigree and the average of the 1000 simulations for each randomization type, using *Z* tests with the calculated standard deviation as described. The distributions of the simulated statistics were good approximations to the normal distribution. *Z* tests were carried out in R version 2.8.1 (R Development Team 2008). Given several *Z* tests were carried out per hypothesis (between 6 and 18), we used a Bonferroni correction to calculate the appropriate significance level.

#### Calculating frequency of re-mating

For each male–female pair known to have mated, we calculated whether they had re-mated when they had the opportunity to do so, giving the number of pairs, females and males that did re-mate and the number which did not, despite having the potential to do so. The potential to re-mate is restricted by the presence or absence of previous partners: in addition to deaths and births changing the available populations of females and males over the study period, in calculating opportunities to mate we also took into account (i) females do not conceive every year and (ii) most males spend the majority of their time resident outside of the study area, only returning for the rut; and not all males known to be alive in the study population return each year. Therefore, for any pair that had mated, we calculated in which other years both (i) the female of the pair was receptive to mating (conceived and gave birth to a calf the following year) and (ii) the male rutted within the study area (and was therefore a potential father in the paternity analysis), and scored whether they re-mated in that year (1 for yes, 0 for no).

From this, we then calculated the number of pairs that had mated in more than 1 year divided by the number of all pairs known to have the opportunity to mate in more than 1 year (as a percentage). We then calculated the percentage of females and males in known pairs that were involved in re-mating events. We also calculated a number of other statistics describing the patterns of re-mating: (i) the average size of full-sibships within the pedigree and (ii) the ratio of different males a female mated with in her lifetime to the number of offspring she produced.

#### Calculating the extent to which female relatives mated with the same male (intralineage polygyny)

We calculated the ratio of different females a male mated with in his lifetime to the number of different matrilines those females came from, so that a value of one describes a male who never mated with females which were relatives, and values < 1 indicate increasing amounts of intralineage polygyny.

#### Relatedness and inbreeding coefficients

Relatedness coefficients were calculated using the R package ‘kinship’ (Atkinson, 2008). Inbreeding coefficients were calculated using the R package ‘pedigree’ (Coster, 2008): we calculated average coefficients, the total number of nonzero coefficients and the number of coefficients ≥ 0.125 (representing close inbreeding events).

### Genetic structuring of the rutting male population

Pairwise relatedness coefficients were calculated for all males in the pedigree. To calculate spatial distances between males, we calculated the lifetime average rutting location of each male to the nearest 100 m from census data, and from this calculated distances between these locations for each pair of males in metres. The correlation between pairwise relatedness and pairwise spatial separation was tested in a linear mixed-effects model, with relatedness as the response variable and the identity of each of the pair as random effects.

## Results

### Re-mating frequency

**Fig. 1:**
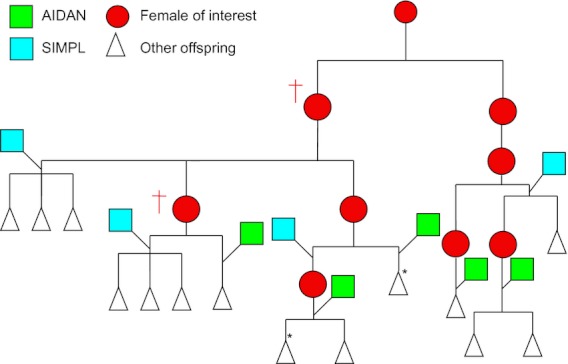
Pedigree illustrating pairs re-mating and intralineage polygyny in matriline 153. Squares refer to males, circles to females that are of interest to this example, and triangles to other offspring not of interest here. The two males shown, ‘AIDAN’ and ‘SIMPL’, can be differentiated by colour or shade. ‘SIMPL’ was involved in several re-mating events, including mating with two females (marked †) in three breeding seasons. ‘AIDAN’ sired both starred offspring; this increased their relatedness coefficient from that of aunt–half-niece (0.125) to aunt–half-niece and half-sibs (*r*: 0.125 + 0.250 = 0.375).

**Fig. 2:**
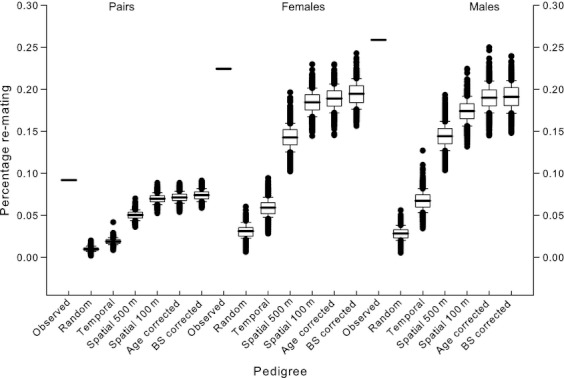
Percentage of pairs, females and males which were involved in at least one re-mating event. Percentages are given for the observed data set and in the simulated pedigrees.

The number of of pairs mated in more than 1 year is 9.2% (134 of 1456), so that 22.4% (109 of 486) of females and 25.9% (60 of 232) of males mated with a partner with whom they had mated previously (see Fig. 1 for an example of this). This was significantly higher than expected under either random mating (‘Random’), random mating constrained to males rutting when a female was in her oestrus window (‘Temporal’), or random mating constrained to males rutting within 500 or 100 m of a female during her oestrus window (‘Spatial 500 m’ and ‘Spatial 100 m’, see Table 1, Fig. 2). The percentage of pairs and males re-mating was also significantly higher in the observed pedigree than in the ‘Age-corrected’ or ‘BS-corrected’ simulations (see Table 1). However, although there was a strong trend towards significance, after a Bonferroni correction, the observed percentage of females re-mating was not significantly greater than in the ‘Age-corrected’ or ‘BS-corrected’ simulations (‘Age-corrected’, *Z* = 2.68, *P* = 0.004, ‘BS-corrected’, *Z* = 2.13, *P* = 0.017, number of tests = 18, therefore, Bonferroni level of significance = 0.003).

**Table 1:** Re-mating frequency in observed pedigree and in randomizations, and comparison. Standard deviations given are for the distribution of percentages from the 1000 runs of the pedigree simulations. *Z* values and *P* values are given for a one-tailed test of the hypothesis that population-level re-mating frequency is significantly higher than would be expected in each randomization. The percentages themselves vary between males, females and pairs because of the different totals of each category in the denominator of the calculation: there are more pairs in total, and fewer individual males than individual females. After a Bonferroni adjustment for the 18 tests within this table, the significance level was taken as α = 0.003

			Comparison to observed re-mating frequency				Comparison to observed re-mating frequency				Comparison to observed re-mating frequency	
									
Model	% Pairs re-mating	SD	*Z*	*P*	% Females re-mating	SD	*Z*	*P*	% Males re-mating	SD	*Z*	*P*
Observed	9.20		N/A	N/A	22.43		N/A	N/A	25.86		N/A	N/A
Full random	0.98	0.25	33.4	< 0.0001	2.84	6.97	28.11	< 0.0001	2.85	0.70	33.04	< 0.0001
Temporal random	1.89	0.33	22.0	< 0.0001	5.93	9.93	16.61	< 0.0001	6.74	1.13	16.95	< 0.0001
Spatial random (100 m)	6.97	0.54	4.1	< 0.0001	18.44	1.30	3.07	0.001	17.43	1.40	6.04	< 0.0001
Spatial random (500 m)	5.02	0.51	8.2	< 0.0001	14.26	1.33	6.12	< 0.0001	14.41	1.40	8.16	< 0.0001
Age-corrected	7.14	0.55	3.7	< 0.0001	18.90	1.32	2.68	0.004	19.01	1.48	4.61	< 0.0001
BS-corrected	7.40	0.60	3.0	0.001	19.45	1.40	2.13	0.017	19.11	1.54	4.39	< 0.0001

BS-corrected, Breeding Success–corrected.

Amongst calves with assigned paternity, on average, females bred in 3.32 ± 0.11 (standard deviation), years, with 3.00 ± 0.09 (standard deviation) different males. In total, 134 parental combinations made up of 108 females and 60 males were repeated on an average of 1.15 ± 0.04 occasions (standard deviation, range 1–3 re-matings). Most re-matings occurred only once; however, four pairs re-mated three times (i.e. mated four times). Re-mating events generally occurred in consecutive years, but some occurred as much as 5 years after the original mating.

As a consequence of re-mating, the average full-sibship size was above one in both the observed data and all simulations. However, full-sibship sizes were significantly higher in the observed pedigree than in any of the simulated pedigrees (see Table 2), indicating none of the processes modelled in the simulations were sufficient to account for the extent of re-mating observed. Further, the ratio of different males a female mated with to the number of calves she produced was also significantly lower in the observed pedigree than in any simulated pedigree (see Table 2).

**Table 2:** A comparison of various statistics describing the pedigree for the observed and each randomized pedigree: full-sibship sizes, the ratio of different males a female mated with over her lifetime to the number of offspring she produced, and the ratio of different females a male mated with to the number of different matrilines those males mated with (intralineage polygyny). For randomized pedigrees, an average and standard deviation were calculated across the 1000 runs of the simulation (no standard deviation is given for the observed value). *Z* test and *P* values are given for a one-tailed test of the hypothesis that (i) full-sibship size is higher in the observed pedigree than in the simulated pedigrees, (ii) the ratio of different males a female mated with over her lifetime to the number of offspring she produced is smaller in the observed pedigree than in the simulated pedigrees and (iii) the ratio of different females a male mated with to the number of different matrilines those males mated with is smaller in the observed pedigree than in the simulated pedigrees. Given for each hypothesis, six tests were carried out, and the significance level after a Bonferroni adjustment was taken as α = 0.008

			Comparison to observed		(Average) ratio different males to offspring produced		Comparison to observed		(Average) ratio different females to matrilines		Comparison to observed	
									
Model	(Average) full-sibship size	SD	*Z*	*P*		SD	*Z*	*P*		SD	*Z*	*P*
Observed	1.106				0.941				0.778			
Full random	1.010	0.003	38.1	< 0.001	0.994	0.002	31.2	< 0.001	0.945	0.004	37.1	< 0.001
Temporal random	1.019	0.003	26.2	< 0.001	0.989	0.002	28.1	< 0.001	0.920	0.005	27.4	< 0.001
Spatial random (100 m)	1.077	0.006	4.8	< 0.001	0.956	0.004	40.8	< 0.001	0.837	0.007	8.1	< 0.001
Spatial random (500 m)	1.054	0.006	9.2	< 0.001	0.969	0.004	4.1	< 0.001	0.858	0.007	11.9	< 0.001
Age-corrected	1.078	0.006	4.4	< 0.001	0.954	0.004	3.4	< 0.001	0.823	0.008	5.85	< 0.001
BS-corrected	1.081	0.007	3.6	< 0.001	0.953	0.004	2.8	0.003	0.823	0.008	5.98	< 0.001

BS-corrected, Breeding Success–corrected.

### Intralineage polygyny

**Fig. 3:**
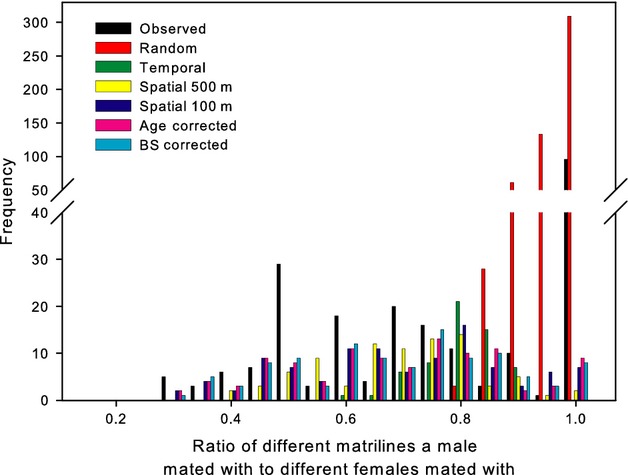
Intralineage polygyny. Frequency histogram of the ratio of the number of different matrilines to which a male's mates belong, to the number of different females the male mated with, in the observed population and simulated pedigrees. Low values therefore indicate more extreme intralineage polygyny. For simulations, an average of the 1000 runs is displayed.

Males mated with females from the same matriline significantly more in the observed population than expected from any of the simulated pedigrees: the ratio of different females a male mated with to the number of different matrilines those females belonged to was significantly lower in the observed pedigree (see Table 2, Fig. 3 and see Fig. 1 for an example) than in any simulated pedigree.

### Relatedness

**Fig. 4:**
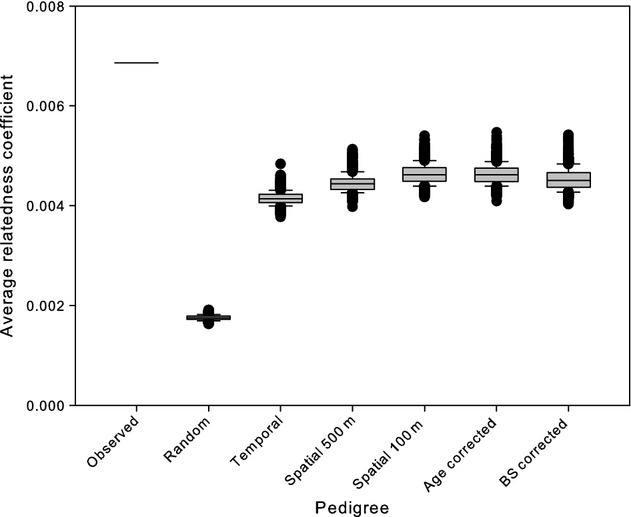
Relatedness coefficients. Comparison of average pairwise relatedness coefficients between individuals in the pedigree in the observed pedigree and in the simulated pedigrees.

On average, pairs of individuals in the observed pedigree were significantly more related than expected under random mating: pairwise relatedness was significantly higher in the observed pedigree than under any simulation (see Table 3, Fig. 4). Figure 1 illustrates how relatedness can be increased as a result of intralineage polygyny (see also discussion).

**Table 3:** A comparison of pairwise relatedness amongst individuals for the observed and each type of simulated pedigree. For simulations, an average and standard deviation for the 1000 iterations of the simulation is given. *Z* tests are presented for a one-tailed test of the hypothesis that the observed value is significantly greater than would be expected from the distribution of simulated values

			Comparison to observed	
			
Model	(Average) relatedness	SD	*Z*	*P*
Observed	0.00687			
Full random	0.00174	0.00005	102.6	< 0.001
Temporal random	0.00415	0.00013	20.9	< 0.001
Spatial random (500 m)	0.00445	0.00017	14.2	< 0.001
Spatial random (100 m)	0.00464	0.00020	11.2	< 0.001
Age-corrected	0.00463	0.00020	11.2	< 0.001
BS-corrected	0.00453	0.00022	37.3	< 0.001

BS-corrected, Breeding Success–corrected.

### Inbreeding

**Fig. 5:**
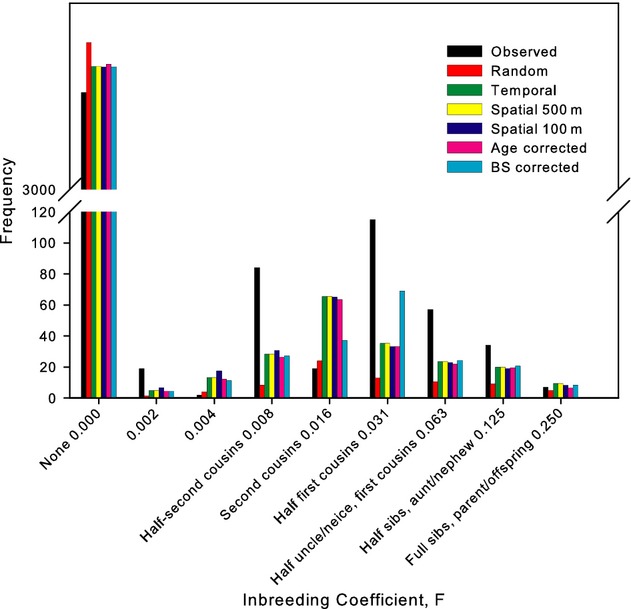
Inbreeding coefficients. Comparison of inbreeding coefficients in the observed pedigree, and the average for each simulated pedigree. Inbreeding coefficients are binned into groups representing key inbreeding events; however, it should be noted that many inbreeding coefficients were intermediate values, due to the effects of intralineage polygyny (e.g. see Fig. 6).

**Fig. 6:**
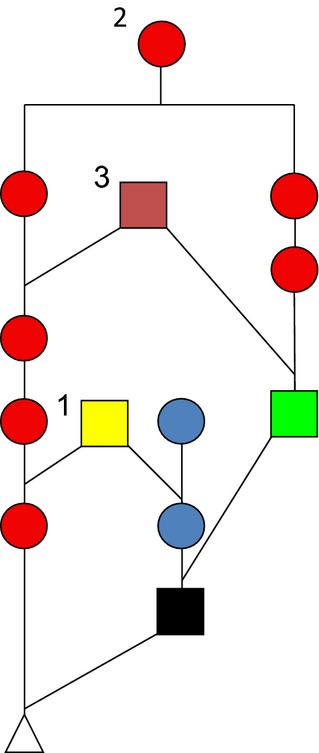
Pedigree illustrating how intralineage polygyny increases inbreeding coefficients. Squares represent males, with different colours or shades representing different males. Females are represented by circles, and the offspring whose inbreeding coefficient is to be calculated is represented as the white triangle. The parents of this offspring are not only aunt–half-nephew (loop 1) and half third-cousins (loop 2); but also, because an aunt and her half-niece both mated with the blue male (loop 3, intralineage polygyny), half first-cousins once removed. Therefore, the inbreeding coefficient for this individual is 0.0625 + 0.0019525 + 0.015625 = 0.08008.

Average inbreeding coefficients were significantly higher in the observed pedigree than in any simulated pedigree (see Table 4, Fig. 5). In addition, the total number of nonzero inbreeding coefficients was significantly higher in the observed pedigree than in any of the simulations (Table 4). We inspected whether this effect was driven by close inbreeding events by determining whether it remained on considering only highly inbred individuals (*f* ≥ 0.125), but it did not: the observed pedigree did not have significantly more close inbreeding events than in the ‘Spatial 100 m’, ‘Spatial 500 m’ or ‘BS-corrected’ simulations and the differences between the observed pedigree and the ‘Temporal’ and ‘Age-corrected’ simulations in the number of close inbreeding events were not significant after Bonferroni correction (see Table 4, Bonferroni significance level *P* = 0.003). This suggests that the increase in average inbreeding coefficients and total number of inbreeding events in the observed pedigree compared to the simulations resulted more from deep inbreeding events (inbreeding between distant relatives) than close inbreeding events. One route by which additional inbreeding events occur is through intralineage polygyny, exemplified by the pedigree in Fig. 6. Inspection of close inbreeding events in the ‘Temporal’, ‘Spatial 100 m’ and ‘Spatial 500 m’ simulations revealed that many of the close inbreeding events in these simulations consisted of mating events between mothers and sons, or half-sibs, in which the males were < 5 years old. These matings, although possible, are unlikely and probably the result of immature males not yet having fully dispersed from the natal group. Therefore, these estimates of close inbreeding coefficients in these simulations are inflated by these unlikely pairings, and so the comparison with the observed pedigree is a conservative one.

**Table 4:** A comparison of inbreeding statistics for the observed and each randomized pedigree: the average inbreeding coefficient, the number of nonzero inbreeding coefficients and the number of coefficients ≥ 0.125. For randomized pedigrees, the mean and standard deviation of the statistic over the 1000 runs of the pedigree simulation are given (no standard deviation is given for the observed value). *Z* test and *P* values are given for a one-tailed test of the hypothesis that the statistic is higher in the observed pedigree than expected from the distribution of statistics calculated from the simulated pedigrees. After a Bonferroni correction, the significance level for the tests in this table was taken as α = 0.003

			Comparison to observed		(Average) number of nonzero coefficients		Comparison to observed		(Average) number of coefficients ≥ 0.125		Comparison to observed	
									
Model	Average inbreeding coefficient	SD	*Z*	*P*		SD	*Z*	*P*		SD	*Z*	*P*
Observed	0.00304				339.00				32.00			
Full random	0.00094	0.00018	11.4	< 0.001	75.77	11.61	22.7	< 0.001	13.01	3.69	5.2	< 0.001
Temporal random	0.00169	0.00022	6.1	< 0.001	217.57	20.09	6.0	< 0.001	20.06	4.56	2.6	0.004
Spatial random (100 m)	0.00204	0.00026	3.9	< 0.001	202.82	22.92	5.8	< 0.001	26.43	4.74	1.2	0.120
Spatial random (500 m)	0.00187	0.00025	4.7	< 0.001	206.21	21.63	6.1	< 0.001	23.81	4.78	1.7	0.121
Age-corrected	0.00177	0.00023	5.5	< 0.001	189.71	21.40	7.0	< 0.001	22.89	4.60	2.0	0.024
BS-corrected	0.00200	0.00026	4.0	< 0.001	204.46	23.62	5.7	< 0.001	25.21	4.66	1.4	0.082

BS-corrected, Breeding Success–corrected.

### Genetic structuring of the male population

**Fig. 7:**
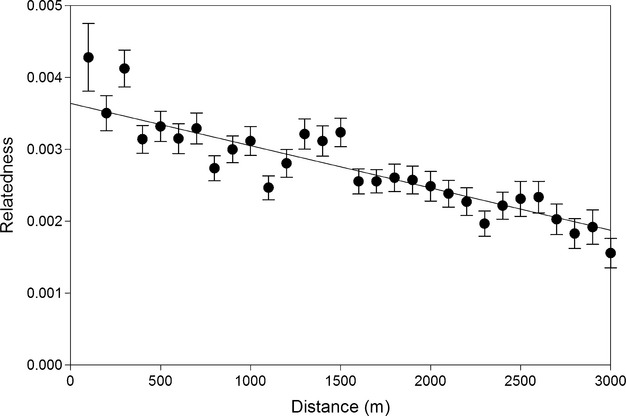
Correlation between relatedness and rutting location of pairs of males. Relatedness of pairs of males plotted against the distance between their average rut locations (pairwise spatial separation).

We found that the location of rutting males was nonrandom with respect to relatedness, so that more closely related males were more likely to rut in the same location: there was a significant negative correlation between male pairwise relatedness coefficients and the pairwise spatial separation (Effect = −1664, *F*_1,174434.4_= 159.23, *P* < 0.001; variance explained by first male identity = 217471 ± 12687 and by second male identity = 119852 ± 4583, see Fig. 7). Note that this analysis is cross-generational; therefore, related males were more likely to rut in the same location regardless of whether they rutted in the same year.

## Discussion

In this study, we have demonstrated that a fifth of red deer females mate with the same male across multiple breeding attempts and that members of the same matriline frequently mate with the same male as one another; in both cases, these events happen more than would be expected under random mating. The observed distribution of matings was associated with a general increase in relatedness across the population compared to that expected under random mating, and also an increase in inbreeding. It should be noted that, like nearly all wild studies, our estimates of inbreeding are likely to be conservative, given the assumption that founders and immigrants are unrelated, and because we cannot assign paternity to all individuals. Although this is also true for the simulated mating scenarios, and the validity of our conclusions is therefore maintained, inbreeding in this population is likely to be even greater than described in this study.

Levels of re-mating and intralineage polygyny observed in the population, and the consequent increase in relatedness and inbreeding coefficients, were greater than expected from the simulated pedigrees in the majority of analyses. In each case, a sequential improvement in ‘fit’ to the observed data was observed as more complexity was incorporated into the simulated pedigrees, so that the simulation incorporating temporal constraints to mating, spatial constraints to mating and a lifetime breeding success–adjusted probability of males gaining paternity success (‘BS-corrected’) most closely predicted observed values in most analyses (with the exception of relatedness and average inbreeding coefficients, in which the ‘Spatial 100 m’ was closest to the observed value). The ‘Age-corrected’ simulation, which incorporated similar levels of complexity, but with the probability of gaining paternity success weighted by male age, was generally also more similar to the observed values than the less complex simulations. This indicates that all of the constraints modelled in the simulations contribute to the extent of re-mating and intralineage polygyny which we have observed: within-individual, and within-matriline, consistency of individuals in their timing and location of rutting behaviour is likely to be important in facilitating re-mating between pairs and probably also intralineage polygyny across years. The further improvement upon adding lifetime breeding success- or age-weighted reproductive probabilities for males probably results because most re-mating events happen in consecutive years, and males have a peak of reproduction which lasts for around 3 years; therefore, if males return to the same rutting locations, the male was likely to have been dominant in that area for consecutive years. A similar argument can be applied to intralineage polygyny, in that female relatives mating in the same place in consecutive years will be likely to mate with the same male; further, within a year, female relatives may be more likely to mate with the same male because he is the most dominant male in the vicinity.

Despite this improvement in fit, significant differences did remain between simulations and the observed pedigree for most parameters, indicating the simulations did not capture the full extent of re-mating and intralineage polygyny. It therefore seems likely that processes not captured by these simulations also affect the distribution of mating. Investigation into the factors affecting the probability of re-mating (Stopher, 2011) suggests that more successful males would be more likely to re-mate with the same female. To an extent, this will be captured in the ‘Age-corrected’ and ‘BS-corrected’ scenarios. However, year-specific male breeding success will be influenced by other factors including environmentally induced variation in antler size and condition (Clutton-Brock *et al*., 1979), as well as socioenvironmental effects on the distribution of male breeding success (Stopher, 2011). Intralineage polygyny may result if female relatives are more likely to mate with the same male because the close association of female relatives means they are likely to be found in the same harem, or possibly, because females copy each other's movements or choice of males. Although this will be captured to some extent in ‘Spatial 100 m’, during the peak of the rut the area captured by this constraint could potentially include a number of harems. Further, females that associate are known to synchronize oestrus (Iason & Guinness, 1985, but note this was not due to kinship *per se*), and the 11-day window we used as a temporal constraint may be too crude to capture this.

Regardless of the extent to which pair re-mating and intralineage polygyny can be explained by the temporal and spatial characteristics of the breeding system, these trends in the distribution of mating are associated with striking effects on the genetic structure of the population. Average relatedness was significantly higher in the observed pedigree than in any simulation. Figure 1 demonstrates why this should be the case where intralineage polygyny exists: in this example, the relatedness of the two individuals is increased from 0.125 (aunt–half-niece) to 0.375, because they also share a father, making them half-sibs. Increased relatedness within groups may promote cooperation between members of the same group (Hamilton, 1963; Griffin & West, 2003; Rossiter *et al*., 2005). Although maternal relatedness more commonly affects affiliation and cooperative behaviour in mammals, there is some evidence that paternal relatedness can also influence the frequency of affiliative interactions between group members (Smith *et al*., 2003; Widdig, 2007). However, to our knowledge, there is as yet no direct evidence that related females show a preference for mating with the same partner.

As shown in Fig. 7, the mating behaviours observed in this study can also result in increased risk of inbreeding. We found that average inbreeding coefficients were greater in the observed pedigree than under any of the random mating scenarios simulated (see Fig. 6). This increase was not driven by an increase in close inbreeding events, as there was no significant difference between the observed and expected in all but the random mating scenario. Instead, it seems likely re-mating and intralineage polygyny have contributed to increased numbers of ‘deep’ inbreeding events, such as that in Fig. 7. Male site fidelity is likely to be important in generating close inbreeding events resulting from intralineage polygyny, such as father–daughter matings. However, deeper inbreeding events may result from male relatives rutting in the same area, particularly males rutting in the same place as their father, that is, within their own natal groups. We have indeed found significant genetic structuring in the male population, indicating that male relatives are likely to be rutting in similar locations. In these cases, the magnitude of the inbreeding coefficient can then be inflated by intralineage events, as occurs in Fig. 7: a relatively distant inbreeding event (aunt–half-nephew) is augmented by an instance of intralineage polygyny higher up the pedigree. In general therefore, it appears a nonrandom distribution of males with respect to relatedness, combined with the mating behaviours we have described, results in an increase in inbreeding in the population over that which would be expected. In many lekking species, males have been shown to exhibit a nonrandom choice of mating sites with respect to relatedness (Petrie *et al*., 1999; Piertney *et al*., 1999; Shorey *et al*., 2000; Höglund & Shorey, 2003), and in grey seals, Pomeroy *et al*. (2000) found evidence males returned to their natal sites to breed, In lekking species, inclusive fitness benefits are generally implicated in such behaviour, as females may be preferentially attracted to larger leks (Shorey *et al*., 2000). Given the short tenure of breeding males, relatives are unlikely to overlap in time as prime-aged individuals and so direct competition is rare. Where direct competition occurs, potentially, in the red deer system, dominant males may be more tolerant of subordinate males near their harem if they are related, but this remains to be investigated. Together, these factors could explain why, to some extent, males return to their natal area to breed, despite dispersing as young males.

Inbreeding is often associated with fitness costs (Keller & Waller, 2002). In this population, there is substantial inbreeding depression for birth weight and first-year survival: a calf with an inbreeding coefficient of 0.25 has a 77% reduction in survival compared to an outbred calf (Walling *et al*., 2011). Why therefore do inbred matings appear to be tolerated in this population? Although many studies have documented fitness costs of inbreeding (reviewed in Keller & Waller, 2002), several reviews have argued that inbreeding should be tolerated where the costs of inbreeding are not greater than the costs of inbreeding avoidance, including costs of dispersal, loss of breeding opportunities or costs of outbreeding, and that such conditions can be realistic (Bateson, 1983; Waser *et al*., 1986; Kokko & Ots, 2006). In particular, much theoretical attention has been paid to the idea that inbreeding tolerance can be favoured by inclusive fitness benefits (Parker, 1979; Smith, 1979; Waser *et al*., 1986; Kokko & Ots, 2006). The benefits of inbreeding in terms of kin selection have been proposed to explain preferences for related males as extra-pair partners in socially monogamous birds (Kleven *et al*., 2005; Wang & Lu, 2011). Inbreeding tolerance is only likely to evolve under such conditions (i) if the male does not lose other breeding opportunities by mating with his kin, which may be true for the red deer system in which male reproductive success is likely to be mostly limited by the ability to gain access to females, rather than time or other ecological constraints; and (ii) the cost of incestuous matings on offspring viability does not outweigh the inclusive fitness benefits of doing so (Smith, 1979; Waser *et al*., 1986; Kokko & Ots, 2006). The increase in inbreeding which we have observed from that expected occurred due to an increase in distant inbreeding events, rather than those between close relatives: therefore, the costs of inbreeding are inevitably reduced. No evidence for inbreeding avoidance has been found in a number of other polygynous systems (Hansson *et al*., 2007; Holand *et al*., 2007; Rioux-Paquette *et al*., 2010). It is not clear whether this is because there is little selection for post-dispersal inbreeding avoidance mechanisms in dispersing species (Clutton-Brock & McAuliffe, 2009) or because the (currently poorly understood) benefits of inbreeding balance, or even outweigh, the costs in such systems. In general, it seems the expectation that animals should always avoid inbreeding requires further thought, and more work remains to be carried out to understand the evolution of inbreeding tolerance or avoidance in such systems.

That this study is conducted on an island population potentially increases the likelihood of the phenomena we have observed: for example, small populations restrict the opportunities for mating and therefore increase inbreeding risk (Keller & Waller, 2002). However, comparison with mainland populations suggests that these phenomena may be more widespread. An investigation of the dispersal of male and female red deer on the Scottish mainland concluded that although dispersal was predominantly male-biased, patterns of relatedness over geographical distances were similar for males and females (Pérez-Espona *et al*., 2008). This study contrasted with the findings of previous work on the Rum population, which showed no spatial genetic structuring of the male population outside of the rut (Nussey *et al*., 2005). However, interestingly, males in the mainland study were sampled during the hunting season (1 July to 20 October), which partly overlaps with the rutting period, the period in which our results indicate spatial genetic structure amongst males in the Rum population.

In summary, using molecular paternity analysis, we have revealed more re-mating between pairs and more intralineage polygyny in a population of wild red deer than expected. Combined with hitherto unquantified genetic spatial structuring of the rutting male population, challenging the assumption of male-biased dispersal in polygynous mammals, these mating behaviours were associated with increased relatedness of individuals in the population, but also an increase in inbreeding events. Such behaviours are rarely documented in wild polygynous mammals, in part because of the challenge of collecting sufficient data across generations to identify them; yet they are key tests of theoretical concepts of population genetics. In general, the combined use of molecular paternity analysis and simulated pedigrees based on potential mating scenarios has revealed further the hidden complexity of this polygynous mating system, and raised many interesting questions for future research: the role of female choice or mate copying, the implications for social evolution and the extent to which inbreeding should be tolerated or avoided in such systems. Identifying, and understanding, such phenomena in wild populations is also critical to wider areas of research: for example, estimates of quantitative genetic parameters, such as trait heritabilities, may be confounded by inflated relatedness amongst closely spatially associated individuals.

## Supporting information

**Data S1** Examining the effect of using categorical pedigrees.

As a service to our authors and readers, this journal provides supporting information supplied by the authors. Such materials are peer-reviewed and may be re-organized for online delivery, but are not copy-edited or typeset. Technical support issues arising from supporting information (other than missing files) should be addressed to the authors.
