# Supplementary material for: Re-mating across years and intralineage polygyny are associated with greater than expected levels of inbreeding in wild red deer
Source: J Evol Biol. 2012 Oct 5;25(12):2457–69. doi: 10.1111/j.1420-9101.2012.02626.x (PMC3546385; doi:10.1111/j.1420-9101.2012.02626.x)
Supplement: Supplementary file 1 [file jeb0025-2457-SD1.doc]

**S1: Examining the effect of using categorical pedigrees**

The use of categorical pedigrees - in which paternities assigned with a specific confidence (in this study, over 80%) are taken as the ‘true’ pedigree, are potentially misleading as they do not incorporate the uncertainty around those paternity assignments.

The program “MasterBayes”, which uses a Bayesian method of parentage assignment (Hadfield *et al.* 2006), calculates many iterations of the potential pedigree, with the point estimate of the pedigree accepting those paternity assignments which appear in 80% of iterations. Instead of calculating a point estimate of the pedigree statistics from the categorical pedigree, by calculating pedigree statistics across many of the Bayesian iterations, an error can be attached to those pedigree statistics, thereby accounting for error in paternity assignments.

**Methods**

The key pedigree statistics - female re-mating frequency, intra-lineage polygyny, inbreeding coefficients and relatedness coefficients - were calculated for each of 1000 iterations of the MasterBayes pedigree, and average values calculated with standard deviations.

The aim of this analysis was to ask whether the discrepancies between the observed pedigree statistics and the simulation data (see main text) could be explained by error around the paternity assignments. Therefore, for each of the key pedigree statistics, we took the distribution of values from the simulation which most closely modelled that seen in the observed pedigree, and compared that to the distribution of values from the 1000 MasterBayes iterations (hereafter known as ‘Observed with error’ pedigrees). The distributions were compared using Wilcoxon tests (non-parametric tests were appropriate because of unequal variances).

It should be noted at this point that the ‘Observed’ pedigree, used in the main text, which uses categorical paternity assignments, is constructed using a combination of MasterBayes and the program “COLONY2” (see main text). Therefore, parameters describing the ‘Observed’ pedigree and ‘Observed with Error’ pedigrees are not directly comparable.

**Results**

*Re-mating frequency of females*

Using the ‘Observed with Error’ dataset, 27.19±0.61% of females were estimated to re-mate with the same male in multiple years. The values of this distribution were significantly greater than the ‘Age Corrected’ simulation (18.90±1.32, W=1000000, p<0.0001), indicating more re-mating was occurring than expected even after accounting for pedigree error.

*Intra-lineage polygyny*

The ratio of unique females a male mated with to matrilines mated with was estimated at 0.807±0.005 for the ‘Observed with Error’ pedigrees. This was significantly lower than in the ‘Age Corrected’ simulation (W=46729, p<0.0001), indicating significantly more intra-lineage polygyny was occurring than in the simulated data.

*Relatedness*

The average relatedness coefficient in the “Observed with Error” dataset was 0.00566±0.00008. This was significantly higher than in the “Spatial 100m” simulation (0.00464±0.00020, W=1000000, p<0.0001). Therefore, pairs of individuals were still more related in this dataset than in the most closely fitting simulation when pedigree error was taken into account.

*Inbreeding*

Average inbreeding coefficients were calculated for the ‘Observed with Error’ pedigrees as 0.00233±0.00108. This was significantly higher than under the simulation scenario which produced the largest average inbreeding coefficients (Spatial 100m, W=851602, p<0.0001). There were significantly more non-zero coefficients in the Observed Error pedigrees (266.92±11.22) than in the Spatial 100m (W=990224, p<0.0001; note Spatial 100m does not have the largest number of non-zero coefficients of all the simulations, which occur in the “Temporal” simulation, but the number is similar, 206.21±21.63 versus 217.57±20.09, and so the comparison is made using Spatial 100m for consistency). However, there were not significantly more close inbreeding events in the ‘Observed with Error’ pedigree (25.57±1.80) than in the Spatial 100m simulation (26.43±4.74, W=448365, p=1.00). Therefore, taking into account pedigree error, our conclusions surrounding inbreeding remain as presented in the main text: inbreeding is greater in the observed population than expected from any simulation, but due to an increase in total non-zero inbreeding coefficients, rather than close inbreeding events.

Hadfield, J. D., Richardson, D. S. & Burke, T. 2006. Towards unbiased parentage assignment: combining genetic, behavioural and spatial data in a Bayesian framework. *Molecular Ecology* **15**: 3715-3730.
